# Design and validation of brucellosis prevention questionnaire focused on animal vaccination

**DOI:** 10.1186/s12889-020-10014-x

**Published:** 2021-01-02

**Authors:** Farhad Bahadori, Fazlollah Ghofranipour, Saeideh Ghaffarifar, Reza Ziaei

**Affiliations:** 1 Department of Health Education, Faculty of Medical Sciences, Tarbiat Modares University, P.O. Box 14115-111, Tehran, Iran; 2 Medical Education Research Center, Health Management and Safety Promotion Research Institute, Tabriz University of Medical Sciences, Tabriz, Iran; 3 Department of Health Sciences, Unit for Public Health Sciences, Mid Sweden University, Sundsvall, Sweden

**Keywords:** Brucellosis, Prevention, Questionnaire, Vaccination, Validity and reliability

## Abstract

**Background:**

The inadequate awareness of livestock breeders on brucellosis transmission, as well as their improper knowledge about preventing brucellosis is considered as one of the important barriers to animal vaccination against brucellosis. The present study aimed to design and validate a brucellosis prevention questionnaire focused on animal vaccination. The valid questionnaire was used to design, implement, and evaluate an interventional training program.

**Method:**

A brucellosis prevention questionnaire (BPQ) was developed in the exploratory psychometric study. In addition, face-to-face interviews were conducted to formulate its initial items, the results of which were merged with those obtained from literature review. Further, the face, content, and construct validity of the questionnaire were assessed by co-operating livestock breeders, veterinarians, and health educationists. The impact score (IS), and content validity ratio (CVR) and index (CVI) of the items were calculated, and the construct validity of the questionnaire was evaluated through factor analysis. Furthermore, the reliability of the results related to the questionnaire was measured by using Cronbach’s alpha, intra-class correlation coefficient (ICC), and composite reliability, as well as the standard error of measurement (SEM).

**Results:**

The questionnaire was finalized with 53 items and its validity was confirmed by CVI (0.90), CVR (0.74), and IS (4.30). Additionally, the items were loaded into three constructs of awareness, attitude, and practice. Further, the predictive power of awareness, attitude, and practice was determined as 43.43, 15.81, and 15.78%, respectively. Furthermore, the fitness of the proposed model among the constructs was confirmed by the root mean square error of approximation (RMSEA) < 0.08, as well as normed chi-square (× 2/ df) < 5.0, comparative fit index (CFI) ≥ 0.90, and Tucker- Lewis index (TLI) ≥ 0.9.

**Conclusion:**

The brucellosis prevention questionnaire represented acceptable psychometric properties. The factors influencing the preventive behavior of livestock breeders can be identified by applying the questionnaire, and co-operating veterinarians and educational planners.

**Supplementary Information:**

The online version contains supplementary material available at 10.1186/s12889-020-10014-x.

## Background

Brucellosis is a highly contagious and zoonotic bacterial disease [1], which a half of the countries with the highest rate of human brucellosis are located in the Middle East [2]. The brucellosis is known as either “Malta fever”, “Mediterranean fever” or “thousand face disease” and has different types. The types of Brucella in Iran include melitensis, abortus, canis, and suis, among which Brucella melitensis is considered as the most widespread and infectious type. The disease is transmitted through contacting infected animals directly or indirectly or consuming their dairy products. In fact, human contact with infected animals is the most common mode of transmission [3].

According to the World Health Organization (WHO) reports, more than 500,000 persons are annually diagnosed with brucellosis worldwide, especially in the developing countries, while about four-fifths of cases are not diagnosed [3]. The results of a systematic review and meta-analysis in 2017 indicated the high incidence of brucellosis in Iran [3]. Iran, as one of the biggest countries in the Middle East, ranks the second with respect to brucellosis prevalence in the world [4]. Based on the results of previous studies, the relative frequency of brucellosis varied from 7 to 276.41 per 100,000 persons in Qom and Kermanshah provinces, respectively. In addition, the west and northwest regions of Iran are considered as the endemic areas for the disease because of observing its highest incidence so that brucellosis is an important health issue in these regions. During the last years, the incidence rate of the disease has reached 130 per 100,000 persons in the west. The inability to control brucellosis in animals is cited as one of the most important reasons for increasing its incidence [3].

The brucellosis, as a disease with thousand faces, is associated with destructive complications and serious disabilities in humans if it is not diagnosed in time. Thus, controlling the disease in livestock and training livestock breeders is considered as significantly important to prevent human infection. The complications of the disease lead to an increase in disability-adjusted life years (DALY) among patients and substantial economic losses to dairy farmers [5]. Animal vaccination decreases the rate of brucellosis infection among persons significantly. Further, WHO recommends the prioritization of animal vaccination to eradicate the disease [6]. Infected animals and their products are considered as the sources of human infection. The disease can be controlled in livestock through vaccinating, killing infected animals and burying their corpses, and quarantining during livestock exchanges. Along with vaccination, public health education is the best approach to prevent the disease among humans [5, 7]. Some of the main barriers to vaccination are related to livestock breeders including their insufficient awareness on brucellosis transmission or improper knowledge about preventing the disease [8]. Due to the absence of effective vaccine for humans, the annual vaccination of sheep and goat with injecting Brucella melitensis vaccine strain Rev.1 is the best possible approach to eradicate Brucella Melitensis in Iran. Vaccination is performed free of charge by the veterinary organization. The organization is considered as the responsible for eradicating the disease through eliminating the contaminated livestock and paying compensation for livestock breeders. In this regard, it performs vaccination and training programs in the country [8]. Based on the studies conducted in Iran, persons are less aware of the transmission ways of the disease [9–11]. Educating livestock breeders is considered as one of the most effective approaches for overcoming the barriers to brucellosis vaccinations [12]. Planning a theory-based and vaccination-focused training program for livestock breeders can be helpful due to the remarkable role of theories and models in designing, implementing, and evaluating the programs [13, 14]. To the best of our knowledge, no theory-driven intervention focused on livestock vaccination has been conducted to prevent brucellosis so far. Consequently, such intervention was designed, performed, and assessed by using a training program for livestock breeders to prevent brucellosis. Additionally, the program should be examined by using a valid and reliable research tool. Designing, implementing, and evaluating the theory-based training intervention, as well as all of the details related to its design based on the four phases of the PRECEDE model, and assessment of its results using reliable and valid tools are fully described in another manuscript (under review).

In the present study, Brucellosis Prevention Questionnaire (BPQ) was applied as the tool, which was introduced thorough an exploratory psychometric study. Further, its design and validation processes, and psychometric properties were presented. In fact, BPQ is considered as a self-administered questionnaire for examining the awareness, attitude, and practice of livestock breeders regarding brucellosis prevention by vaccine. Veterinarians and educational planners can identify the factors affecting the preventive behavior of livestock breeders by using BPQ, as a valid and reliable questionnaire.

## Method

### Research design, context and participants

The present exploratory psychometric study was conducted in Beiragh during 2018 after receiving approval from the Ethics Review board at Tarbiat Modares University. Beiragh village is located in the northern slope of Sahand Mountain in the south of Tabriz metropolis. About 5000 persons live in Beirgh, the employment and economic growth of most of whom depend on their livestock. In addition, livestock husbandry is considered as the main job of Beiraghians so that most of dairy products in the country are produced in villages such as Beiragh. The livestock of Beiraghians mainly included sheep and goats and rarely cows, which are vaccinated by two veterinarians and two livestock vaccinators in each year with paying no charge.

Table 1 summarizes the details related to the participants in each phase, which indicates the recruitment of different participants depending on the study phase.

**Table 1:** Details for the participants in each phase of the study

Phase of study	Participants’ job and number male(m) + female(f)				Assessment measure or target
	Livestock breeder	Health educationist	Veterinarian	Experts from a vaccine providing institute	
**Item generation**					
Interviews	6 m	4(1 m + 3f)	4(3 m + 1 f)	3 m	Identifying the factors influencing preventive behavior of livestock breeders
Group discussion	0	3f	3 m	3 m	Finalizing the first draft of the research questionnaire
**Assessment of face validity**					
Qualitative way	15 m	7(2 m + 5f)			Resolving ambiguity in meaning, wording, grammatical errors and allocation of the items
Quantitative way	15 m	0	0	0	Calculating IS of the items
**Assessment of content validity**					
Qualitative way	0	7(2 m + 5f)	7(5 m + 2f)	3 m	Calculating CVR of the items
Quantitative way	0	7(2 m + 5f)	7(5 m + 2f)	3 m	Calculating CVI of the items
**Assessment of construct validity**					
EFA	212 m	0	0	0	Calculating KMO of the questionnaire; factor loadings
CFA	220 m	0	0	0	Calculating fit indices of the structural behavioral model
**Reliability assessment**	42 m	0	0	0	Calculating Cronbach’s α, ICC,SEM,CR of the questionnaire

Selecting specialists with valid articles or work experience in the intended field is considered as important. Therefore, the experts were invited in the study from different groups with the above-mentioned qualifications to help generate items, finalize the first draft of the questionnaire, and evaluate the face and content validity of the items [15, 16]. Further, livestock breeders were requested to participate in formulating items and examining their face and construct validity. Furthermore, all of the livestock breeders were male and the details of their selection in each phase are described as follows.

### Developing the first draft of the questionnaire

The initial items of BPQ were achieved through a thorough literature review and interview with all stakeholders. Additionally, the databases including MEDLINE, PubMed, EMBASE, ERIC, and Cochrane Library, as well as the Cumulative Index of Nursing and Allied Health Literature (CINAHL) were searched to find the studies published about brucellosis prevention or animal vaccination. In this regard, a combination of the keywords of “prevent”, “Brucella” and “vaccine” were utilized to explore in English and Persian. Thus, 816 articles issued between 2008 and 2019 were obtained, their abstracts were read, and duplicate ones were removed, of which 110 more relevant ones were read in full text. Then, nine Persian and three English questionnaires were obtained by reviewing the articles and contacting corresponding authors [17–19].

Interviews were conducted to identify the factors influencing preventive behavior of livestock breeders. The conceptual framework for conducting the interviews was the concepts from the first four phases of PRECEDE model [14]. Purposive sampling was employed to recruit participants [20] and directed content analysis was used to analyze the content of the interviews [21].

The volunteer livestock breeders, health educationists, veterinarians, and experts from a vaccine and serum production institute in the region participated in 30–45-min face-to-face interviews in their desired time and place. They were told that their information would be kept confidential and used anonymously. The items obtained from literature review were combined with the results of interviews. In addition, health educationists, veterinarians, and experts from a vaccine and serum production institute were asked to participate in three focus group discussions. Each session lasted 90 min, one member of the research team acted as the coordinator of the sessions, and another took note. Then, a directed analysis was performed on the content of the interviews, identical and duplicate questions were eliminated, and some questions were edited. Finally, the first draft of the study questionnaire was confirmed, and the anchor response of the items were discussed and finalized by the research team members.

### Assessment of face and content validity of the questionnaire

The face validity of the intended questionnaire was examined qualitatively and quantitatively by co-operating livestock breeders and health educationists. In qualitative evaluation, any ambiguity in the meaning, wording, and scaling of the items, as well as grammatical errors and those in item allocation were identified and resolved based on the feedback from livestock breeders and health educationists. However, the impact score (IS) of each item was calculated for quantitative assessment.

The livestock breeders participating in the evaluation differed from those involved in examining the construct validity of BPQ and those co-operating in the cross-sectional part of the study.

In order to assess the face validity of the items, the appropriateness of each item was rated by an expert by using a five-point Likert scale, and the IS of each item was calculated by using the formula of.

IS = frequency (%) × importance [22, 23]*.* In the fourmula, the frequency represents the number of the patients rating the appropriateness of the item as 4 or 5, while importance refers to the mean score of the item on a 1-5scale.

Additionally, the content validity of BPQ was evaluated qualitatively and quantitatively. In this regard, content validity index (CVI) and ratio (CVR) were calculated for quantitative assessment. To this end, BPQ was emailed to 20 veterinarians and health educationists for evaluating the validity. One expert failed to complete the questionnaire and two questionnaires were set aside by considering the precision of the data. (Response rate = 0.85%).

The CVI and CVR were determined based on the three and four-point Likert scales, respectively. The formula of (Ne – N/2)/ (N/2) was used to calculate CVR [23], in which N indicates the total number of panelists and Ne illustrates the number of those rating the item as “essential”. Further, items with CVR below 0.46 were removed based on the Lawshe table [24].

In order to compute the CVI of the items, the relevance of each item was rated in a four-point Likert scale by using the formula of CVI (the number of the specialists who assigned scores 3 and 4 to the items/N). Furthermore, the items with the CVI less than 0.79 were eliminated [25, 26].

### Assessment of construct validity of the questionnaire

The construct validity of BPQ was examined through exploratory factor analysis (EFA) and confirmatory factor analysis (CFA). It is recommended to perform each of EFA or CFA by participating at least 200 ones from the target group [27]. Sampling framework in the phase of the study included 2122 livestock breeders living in Beiragh. Since livestock breeders deliver their dairy products to the local dairy production mini-factories in the region, the complete list of livestock breeders was prepared from forty cheese production mini-factories in the village. Due to 10–15% drop rate in the previous relevant studies, there was a need for 50 more participants [27, 28]. In the phase, 450 livestock breeders were randomly selected from Beiragh and invited for participation by using (www.randomizer.org software (.

### Exploratory factor analysis (EFA)

EFA was implemented on 42 binary items and 17 Likert-scale ones, which were intended to explain the preventive behavior of livestock breeders. In addition, the number of optimal factors was determined through principal component analysis and oblimin rotation method. Loadings with the significance lower than 0.5 were excluded from the analysis [29]. If an item was loaded into different factors, it was related to the factor in which the item had the largest factor loading. After completing the analysis, the items were categorized and each category formed a construct or factor. Then, the extracted factors were named by team members based on the nature of their items, as well as the characteristics proposed by them to measure.

### Confirmatory factor analysis (CFA)

For the items with binary response anchors, the generalized confirmatory factor analysis [30] and WLSMV statistical estimation method were applied for the items with binary response anchors [30]. Additionally, the intended conceptual model was tested by using M-Plus 7.4 software. A conceptual model was proposed to test by considering the factors recognized by the EFA and hypothetical relationships between the factors. Further, 59 items were grouped into three factors of awareness, attitude, and practice. Due to the improper fitness of the initial three-factor conceptual model, the awareness was divided into direct, indirect, and vaccine awareness. Furthermore, the suggested new conceptual model with 59 questions and 5 latent factors (direct, indirect, and vaccine awareness, attitude, and practice) was evaluated through confirmatory factor analysis. After removing the items having low factor loadings, the final model with five factors (behavioral constructs) and 53 items was confirmed.

The fitness of the proposed model was assessed by using the fit indices including the ratio of chi-square to the degrees of freedom (X^2^/DF) and root mean square error of approximation (RMSEA), as well as comparative fit (CFI) and Tucker-Lewis indices (TLI). The CFI and TLI at least 0.90 and RMSEA below 0.08 represent a good fitness [31, 32]. The final conceptual model was introduced after excluding non-significant items.

### Assessment of reliability of the questionnaire

The internal consistency of BPQ was tested by calculating Cronbach’s alpha coefficient and composite reliability (CR). In addition, the stability of the results was evaluated by determining interclass correlation coefficient (ICC) [23, 24]. Due to the need for about 30–40 participants to assess reliability [33], 42 volunteer livestock breeders completed the questionnaire twice in a two-week interval, and consequently the stability of the results was measured [34]. Further, the standard error of measurement (SEM) was calculated to analyze the absolute reliability of the results. Furthermore, IBM SPSS statistics version 24 was utilized to perform data cleaning and compute reliability indices. *P* values less than 0.05 were considered as significant.

## Results

After completing the validation process, some items were removed. The final research questionnaire included 10 questions about baseline characteristics of the participants and 59 content-specific questions.

### Baseline characteristics of the participants

The mean age of the participants was obtained as 51.68 ± 16.40 years. Table 2 represents the baseline characteristics of the participants, which were divided based on the each phase of the study.

**Table 2:** Baseline characteristics of livestock breeders divided by each phase of construct validity of the research questionnaire

Characteristics	Total (***n*** = 432)	Exploratory Factor analysis data (***n*** = 212)	Confirmatory Factor analysis data(***n*** = 220)	***P***-value^a^
	Number (%)	Number (%)	Number (%)	
**Age: Mean ± SD)**	51.68 ± 16.40	51.43 ± 16.78	51.92 ± 16.05	0.76
**Sex**				
Male	432(100%)	212(100%)	220(100%)	
Female^b^	0(0%)	0(0%)	0(0%)	
**Animal type**				0.77
Cow	53 (12.3%)	25 (11.8%)	28 (12.7%)	
Sheep & Goat	379 (87.7%)	187 (88.2%)	192 (87.3%)	
**Job**				0.91
self-employed	16 (3.7%)	6 (2.8%)	10 (4.5%)	
employee	12 (2.8%)	6 (2.8)	6 (2.7%)	
Farmer	329 (76.1%)	162 (76.4%)	167 (75.9%)	
Unemployed	75 (17.4%)	38 (18.0%)	37 (16.9%)	
**Educational level**				0.95
Illiterate	191 (44.2**%**)	92 (43.4%)	99 (45.0%)	
Elementary	14 (42.6**%**)	93 (43.9%)	91 (41.4%)	
High school	48 (11.1**%)**	23 (10.8%)	25 (11.4%)	
Graduate diploma	9 (2.1**%**)	4 (1.9%)	5 (2.3%)	
**Income**				0.64
Less than household expenses	382 (88.4%)	189 (89.2%)	193 (87.7%)	
Equal to household expenses	50 (11.6%)	23 (10.8%)	27 (12.3%)	
**Prior brucellosis prevention**				
No	412 (95.4%)	202 (95.3%)	210 (95.5%)	0.93
Yes	20 (4.6%)	10 (4.7%)	10 (4.5%)	
**Previous history of brucellosis in humans**				0.79
No	256 (59.3%)	127 (59.9%)	129 (58.6%)	
Yes	176 (40.7%)	85 (40.1%)	91 (41.4%)	
**The number of family members** (Range)	7 (2–12)	6 (3–11)	7 (2–12)	0.96

^a^The relationship between the two nodes of exploratory and confirmatory factor analysis

^b^ All livestock breeders in this region were male

### Results of the face validity of the BPQ

All items of BPQ became clear and understandable. The impact score (IS) of items ranged between 3.6 and 4.8. IS of all items is shown in Table 3.

**Table 3:** The measures of face and content validity of the items of brucellosis prevention questionnaire (BPQ)

Construct or sub-construct		Item	Content Validity Ratio	Content Validity Index	Impact Score	The source^a^ of the items (Literature/ interviews
**AD = direct awareness score**	1	Can brucellosis be transmitted from an animal to a human?	0.88	1	4.8	L
	2	Can brucellosis be transmitted from a sheep or a goat to a person?	0.64	0.98	4.8	L
	3	Can brucellosis be transmitted from a cow to a human?	0.76	0.96	4.7	L
	4	Is brucellosis transmitted from a person to another?	0.76	0.86	4.4	L
	5	Can brucellosis be transmitted from skin contact with an infected animal?	0.88	1	4.2	L
	6	Can brucellosis be transmitted by breathing into the infected stalls of livestock?	0.64	0.84	4.7	L
	7	Can brucellosis be transmitted through touching the aborted fetus and placenta of an infected animal?	1	0.94	4.7	L
	8	Can wearing gloves prevent brucellosis when you contact the uterine secretions of dead animals?	0.64	0.80	4	L
	9	Does brucellosis spread in the environment by the urine of animal?	0.88	0.90	3.8	L
	10	If your animal aborts its fetus, do you burn the aborted fetus?	0.64	1	4.1	L
	11	Does brucellosis spread in the environment by the’ fetus and placenta of animal?	0.76	0.94	4.2	I
	12	Does the brucellosis spread in the environment by wool?	0.64	0.98	4.3	L
	13	Does killing infected animals prevent the development of brucellosis?	0.52	0.94	3.8	L
	`14	Do you think that abortion place should be disinfected during the abortion?	0.76	1	4.7	L
	15	Do you think a dog can eat an aborted fetus?	0.52	0.80	4.2	I
	16	Do you think the aborted fetus needs to be buried?	0.52	0.86	3.8	I
	17	Is brucellosis a preventable disease?	0.88	0.80	4.7	L
	18	Should the place be disinfected after an abortion?	0.76	0.80	4.2	L
**AID = Indirect awareness**	1	Can brucellosis be transmitted through consuming infected milk and dairy products?	0.64	0.90	4	L
	2	Can brucellosis be transmitted by semi-cooked meat?	0.76	0.88	4.2	L
	3	Can brucellosis be transmitted through breathing into the stalls of animals?	0.88	0.92	4.1	I
	4	Can washing the milking dishes prevent brucellosis?	0.52	0.80	3.7	I
	5	Can boiling milk prevent brucellosis?	0.76	0.80	3.6	L
	6	Can keeping cheese in salty water for two months before consumption prevent brucellosis?	0.76	0.86	4.2	L
	7	Does the use of a mask while cleaning the stable prevent Brucella disease from transmitting?	0.64	0.90	3.8	L
	8	Does brucellosis spread in the environment by the milk of infected livestock?	0.88	0.92	4.7	I
	9	Does brucellosis spread in the environment by the meat of infected livestock?	0.64	0.94	4.6	I
	10	Do you ask a veterinarian to help with animal abortion?	0.52	0.98	4.1	I
**AV=Vaccine oriented behavior score**	1	Can livestock vaccination prevent Malta fever among humans?	0.52	0.92	4.7	L
	2	Can brucellosis be prevented by vaccinating livestock?	0.76	0.80	4.2	I
	3	Is animal vaccination expensive for you?	0.88	0.90	4.3	I
	4	Do you have access to livestock vaccination services?	0.88	0.82	4.7	I
	5	Does the veterinary organization offer timely vaccination services for your animals?	0.64	0.96	4	I
	6	Do veterinarians encourage you to vaccinate your livestock??	0.64	0.98	4.2	I
	7	Do the health personnel encourage you to vaccinate your livestock?	0.76	0.94	4.4	L
	8	Do family members encourage you to vaccinate your livestock?	0.64	1	4.1	I
	9	In which season should brucellosis vaccine be injected?	0.88	0.84	4.7	L
	10	How often should the brucellosis vaccine be repeated?	0.64	0.96	4.8	I
	11	I would like to prevent the disease through vaccination since the veterinarian organization does not pay full compensation for the animals slaughtered due to brucellosis.	0.52	0.82	4.2	L
	12	At what age should livestock be vaccinated against brucellosis?	1	0.98	4.8	L
	13	Does brucellosis affect livestock growth after vaccination?	0.88	0.80	4.2	I
	14	Can livestock get brucellosis again after vaccination?	0.52	0.82	4	I
**A = Attitude score**	1	Abortion does not occur if animals are vaccinated.	0.76	0.84	4.1	L
	2	Livestock breeders should ask a veterinarian to examine their livestock.	0.88	0.98	3.9	I
	3	I will not get brucellosis, if I touch Vaccinated animals’ milk, urine, placenta and fetus.	0.76	0.94	4.6	L
	4	As vaccination is time-consuming, I prefer getting brucellosis instead of vaccinating my animals.	0.88	0.92	4.8	L
	5	Animal vaccination is a very difficult process.	0.52	0.96	4.2	L
	6	If I get brucellosis, I will be unable to work for a long time.	0.76	1	3.2	L
	7	If I do not vaccinate the animals, my family and I will get brucellosis.	0.88	0.82	4.7	L
	8	If I vaccinate my livestock, people will not get brucellosis with consumption of my dairy products.	0.76	0.86	4.8	L
	9	I may still be in danger of getting brucellosis, even if I do preventative measures.	0.88	0.98	4.2	I
**P = practice score**	1	I keep the records and documents related to the medical history and vaccination of my animals in a proper place.	1	0.94	4.8	I
	2	I have experienced abortion among my livestock.	0.52	0.84	4.2	L
	3	I always monitor the cold chain for the vaccines injected into my cattle.	0.64	0.96	4.8	L
	4	I vaccinate my animals at the proper and recommended time in a year or season.	0.88	0.98	4.7	L
	5	I have vaccinated my livestock against brucellosis in every year since many years ago.	0.76	0.88	4.1	L
	6	My livestock are in contact with other non-vaccinated livestock.	0.64	0.82	3.8	L
	7	During buying a new livestock, I am curious to know about its vaccination history.	0.88	0.80	4.2	I
	8	During buying a new animal, I ask a veterinarian to examine my animal.	0.76	0.80	4	I
**Mean**			**0.74**	**0.90**	**4.3**	

^a^literature(L) or interview(I)

### Results of the content validity of the BPQ

Table 3 demonstrates the CVI and CVR of the items, which indicates their ranges as 0.80–1.00 and 0.52–1.00, respectively.

### Results of the construct validity of the BPQ

Among 450 completed questionnaires, 18 incomplete ones were excluded, and EFA and CFA were respectively implemented based on the 212 and 220 completed ones.

### Results of the exploratory factor analysis

The generalized EFA was conducted due to the binary measurement level of the items. In fact, EFA is used to determine the number of the continuous latent variables needed to explain the correlations among a set of binary observed ones. The factors and their indices were respectively considered as continuous latent and observed variables. In the study, the factors were extracted by using maximum likelihood estimation method and rotated using oblique GEOMIN rotation procedures. Additionally, Likert-type items were loaded into two factors of attitude (9) and practice (8). Further, binary-type items were loaded in a factor including three sub-scales of awareness about the direct transmission pathways (AD) Awareness about indirect transmission pathways (AID) and Vaccine-oriented awareness (AV), with 16, 9 and 12 items respectively. The variations in the preventive behavior of livestock breeders were predicted by changing their awareness, attitude, and practice scores. The predictive power of awareness, attitude, and practice scales was 43.43, 15.81, and 15.78%, respectively. Table 4 summarizes the loadings of the extracted factors.

**Table 4:** The results of measurement model divided by each construct of the brucellosis prevention questionnaire (BPQ)

Scale	χ2	χ2/df	P	CFI	TLI	RMSEA (95% CI)	Eigenvalue	%variance	Cumulative % variance
Direct Awareness (AD)	270.20	2.00	< 0.001	.940	.932	.069 (.057; .081)	9.41	15.89	15.89
Indirect Awareness (AID)	42.36	1.21	0.183	.990	.987	.031 (.001; .061)	4.93	14.81	30.70
Vaccine oriented Awareness (AV)	235.87	3.06	< 0.001	.976	.953	.079 (.064; .093)	5.65	12.73	43.43
Attitude	44.40	1.64	0.019	.991	.988	.055 (.023; .083)	5.26	15.81	59.24
Practice	91.6	4.58	< 0.001	.972	.961	.079 (.053; .106)	4.67	15.78	75.02

χ2, chi-square; χ2/df, normed chi-square; CFI, comparative Fit Index; TLI, Tucker and Levis Index; RMSEA, root mean square error of approximation

### Results of the confirmatory factor analysis

**Fig. 1:**
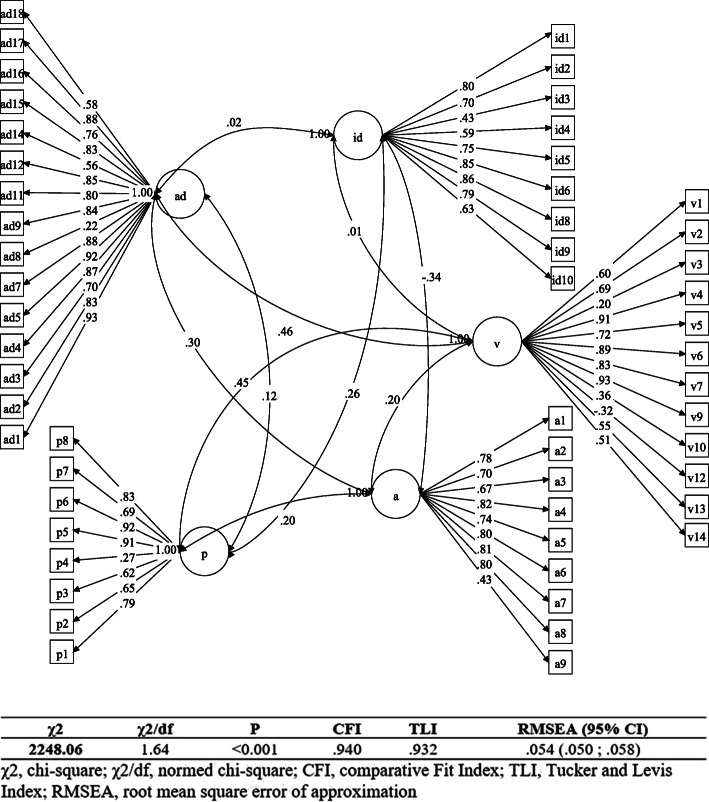
Routing diagrams for generalized validation model for all structures

Confirmatory factor analysis for the 17 Likert-type items and 37 binary items was done. In this process of analysis, 53 remaining items were considered as indicators and 5 latent variables were considered as the constructs. All the relationships between the constructs (AD, AID, AV, A, and P) and items (or 53 indicators) were significant (All *P* < 0.05). The results indicated a good fitness of the model. The model and the loadings of the extracted factors are presented in Fig. 1.

Additional results of CFA, including factor loadings and T-statistics were reported in Table 5.

**Table 5:** The results of the final confirmatory factor analysis of the Brucellosis Prevention Questionnaire (model factor loadings and T-values)

	Direct Awareness (AD)		Indirect Awareness (AID)		Vaccine oriented Awareness (AV)		Attitude		Practice	
Items	loading	t-value	loading	t-value	loading	t-value	loading	t-value	loading	t-value
**AD1**	0.925	27.870								
**AD2**	0.834	23.533								
**AD3**	0.702	12.375								
**AD4**	0.866	34.158								
**AD5**	0.917	35.040								
**AD7**	0.884	26.580								
**AD8**	0.222	3.568								
**AD9**	0.842	34.573								
**AD11**	0.799	16.714								
**AD12**	0.848	15.297								
**AD14**	0.565	11.379								
**AD15**	0.831	25.834								
**AD16**	0.758	11.966								
**AD17**	0.884	36.951								
**AD18**	0.578	9.986								
**ID1**			0.802	12.538						
**ID2**			0.705	16.687						
**ID3**			0.427	7.036						
**ID4**			0.587	12.442						
**ID5**			0.754	26.469						
**ID6**			0.852	17.374						
**ID8**			0.856	30.126						
**ID9**			0.793	16.429						
**ID10**			0.628	9.095						
**AV1**					0.599	11.516				
**AV2**					0.692	15.700				
**AV3**					0.205	3.062				
**AV4**					0.910	32.667				
**AV5**					0.718	20.175				
**AV6**					0.891	22.625				
**AV7**					0.832	23.891				
**AV9**					0.926	21.381				
**AV10**					0.356	6.135				
**AV12**					−0.321	−3.502				
**AV13**					0.554	10.857				
**AV14**					0.508	11.044				
**A1**							0.779	31.494		
**A2**							0.700	19.999		
**A3**							0.673	21.465		
**A4**							0.822	36.302		
**A5**							0.738	23.219		
**A6**							0.799	34.801		
**A7**							0.808	35.827		
**A8**							0.802	34.834		
**A9**							0.427	9.326		
**P1**									0.788	29.746
**P2**									0.653	19.495
**P3**									0.618	20.255
**P4**									0.270	5.403
**P5**									0.912	57.672
**P6**									0.924	62.573
**P7**									0.693	21.539
**P8**									0.830	32.831

Based on the findings from participation of 432 livestock breeders, summary of the descriptive statistics of the KAP analysis of the psychometric study of the Brucellosis Prevention Questionnaire (BPQ) is shown in Table 6.

**Table 6:** Descriptive statistics of the KAP analysis of the psychometric study of the Brucellosis Prevention Questionnaire (BPQ) (n = 432 livestock breeders)

Scale	Number of Questions in scale	Mean ± SD	Range
**Awareness in all**	36	14.71 ± 5.79	1–31
**Direct Awareness (AD)**	15	3.36 ± 3.60	0–15
**Indirect Awareness (AID)**	9	6.04 ± 2.50	0–9
**Vaccine oriented awareness (AV)**	12	5.30 ± 2.87	0–11
**Attitude**	9	25.46 ± 7.26	11–45
**Practice**	8	17.42 ± 7.90	8–39

### Results of the reliability of the questionnaire

The measures of reliability, divided by each scale of the research questionnaire are presented in Table 7.

**Table 7:** The measures of reliability, divided by each scale (factor) of the Brucellosis Prevention Questionnaire (BPQ)

Construct or sub = construct	Intra-class Correlation Coefficient ICC (95% CI)	Cronbach’s Alpha (α)	standard error of measurement (SEM)	Composite Reliability (CR)
**Awareness in all**	0.958(0.920–0.978)	0.865	5.448	0.974
**Direct Awareness (AD)**	0.957(0.919–0.977)	0.863	7.591	0.951
**Indirect Awareness (AID)**	0.947(0.900–0.972)	0.831	10.446	0.908
**Vaccine oriented awareness (AV)**	0.885(0.783–0.939)	0.718	11.330	0.895
**Attitude**	0.896(0.803–0.945)	0.833	8.231	0.912
**Practice**	0.927(0.863–0.962)	0.825	10.477	0.896

The final BPQ is attached as Additional file 1. The file represents all constructs and item of BPQ as a valid and reliable questionnaire.

## Discussion

The BPQ is considered as the first theory-based questionnaire focused on animal vaccination, which was designed and validated for using in the interventions for preventing brucellosis. Some researchers studied the effect of training on brucellosis prevention among livestock breeders by using researcher-made questionnaires, while the psychometric properties of the questionnaires were not reported [12, 17, 35–38]. BPQ has acceptable psychometric properties and all of its items were loaded into awareness, attitude, and practice constructs. In addition, 75% of changes in the brucellosis preventive behavior of livestock breeders were predicted by the variations in their awareness, attitude, and practice scores. Further, the predication power of awareness, attitude, and practice constructs were determined as 43.43, 15.81, and 15.78%, respectively.

In the present study, awareness was the most predictive construct of the preventive behavior of livestock breeders. Some research highlighted the role of awareness and attitude in changing health behaviors [12, 17, 39]. For instance, the attitude and practice scores of livestock breeders improved significantly after a PRECEDE-based training intervention based on the results of a quasi-experimental study [38]. Furthermore, the psychometric properties of the tool were not assessed completely and only Cronbach’s Alpha was reported for the predictive constructs of awareness (0.83), attitude (0.8), and practice (0.9) [38].

The items of the BPQ were internally consistent. In the present study, the Cronbach’s Alpha for awareness, attitude, and practice was determined as 0.865, 0.833, and 0.825, respectively, which indicates the acceptable internal consistency of the items [40].

The fitness indices of the final conceptual model in the study were considered as proper (χ2 = 2248.06; χ2/df = 1.64; RMSEA = 0.054; CFI = 0.940; TLI = 0.932) since the fitness of structural equation modeling is confirmed when RMSEA is below 0.08 and CFI and TLI are above 0.9 [31].

Based on the results, CVI and CVR in the items of the questionnaire ranged between 0.80–1.00 and 0.52–1.00, respectively, which confirms its ability to assess intended cases [24–26].

Items of the final BPQ had impact scores above 1.5. Impact scores of the items ranged between 3.6 and 4.8. These findings represented good face validity of BPQ [41].

Further, the reliability of the results was confirmed by the ICC (at least 0.885), CR (0.895), and SEM (5.448) of the constructs, which demonstrates the acceptable internal consistency and stability of the results related to BPQ [22, 42].

## Conclusions

Based on the results of the study, the designed BPQ is considered as a valid and reliable questionnaire, which can be used to evaluate the effectiveness of training interventions. In general, other health educationists and researchers can identify the effective factors on the preventive behavior of livestock breeders by employing the BPQ. Co-operating the sectors, and public and governmental institutions is required for preventing a disease with a high prevalence across the world, especially in the Middle East and different transmission ways. Other issues such as economic and cultural can influence the process, which requires further studies. However, training interventions are considered as effective and necessary to prevent and eradicate the disease.

### Strengths of the study

BPQ is the first theory-driven questionnaire in the field of brucellosis prevention and focuses on real problems, barriers and facilitators of preventive behavior of livestock breeders. It was designed and validated for a theory-driven and vaccination-focused training program. In this study, triangulation method was employed to generate the items of the research questionnaire. The items were produced by literature review, the main researcher’s field notes and face-to- face interviews with different stakeholders (livestock breeders, health educationists, veterinarians and experts from vaccine and serum production institute).

### Limitations of the study

In this study, 44% of the livestock breeders were uneducated. The main researcher had to explain all questions to the participants and complete the research questionnaires by himself.

Each sentence and question was explained in the language of the livestock breeders and the answers were checked with the livestock breeders himself. If one of the family members was literate, the forms would be completed in the presence of a literate person. This required more time for explanation for each participant. Also, due to the fact that in Iran, vaccination is provided for free by veterinary organization, the cost consideration and its effects on preventive measures have not been investigated in this study. Moreover, providing gloves or sanitizer liquids, etc. are costly and not affordable by our livestock breeders; therefore, we couldn’t address these issues in our research.

### Implications

The BPQ, which was designed and validated in this study, can be employed by all health educationists to plan for necessary educational interventions.

## Supplementary Information

**Additional file 1.** Brucellosis Prevention Questionnaire (BPQ). The final BPQ is attached as Additional file 1. The file represents all constructs and item of BPQ as a valid and reliable questionnaire.

## Data Availability

All data and materials will be available on reasonable request from the corresponding author.

## Abbreviations

BPQ: Brucellosis Prevention Questionnaire
CFI: Comparative Fit Index
CVI: Content Validity Index
CVR: Content Validity Ratio
ICC: Intra-class Correlation Coefficient
RMSEA: Root Mean Square Error of Approximation
SRMSEA: Standardized Root Mean Square Error of Approximation
TLI: Tucker- Lewis Index
