# Supplementary material for: Design and validation of brucellosis prevention questionnaire focused on animal vaccination
Source: BMC Public Health. 2021 Jan 2;21:2. doi: 10.1186/s12889-020-10014-x (PMC7777352; doi:10.1186/s12889-020-10014-x)
Supplement: Supplementary file 1 — Additional file 1. Brucellosis Prevention Questionnaire (BPQ). The final BPQ is attached as Additional file 1. The file represents all constructs and item of BPQ as a valid and reliable questionnaire. [file 12889_2020_10014_MOESM1_ESM.docx]

Completion date of the questionnaire: ID number of your workplace:

| Dear Livestock Breeder  The following questionnaire has been designed to investigate the effect of an educational intervention on brucellosis prevention and livestock vaccination process. Your cooperation will help us to take useful steps in improving brucellosis prevention-related behaviors. Surely, the information will be kept completely confidential. They will be used for only research work. Really appreciate your invaluable time and cooperation in completing this questionnaire. |
| --- |

**Age** (year):

**Gender**: Female  Male

**What kind of livestock are you breeding?** Cow  Sheep & Goat 

**What else do you do in addition to livestock breeding?**

Self-employed  employee  Farmer Unemployed 

**Education level:**

Illiterate  Elementary  High school Graduate diploma 

**Have you ever been trained about brucellosis prevention?** Yes No 

**If yes, where did you receive that training**? Training Classes 

Local health care center 

**Have you ever had a history of brucellosis in yourself or in your family members?**

Yes No 

**If yes, how long ago?**

**Number of family members you are living with**:: ...............

**Responding to questions from 1 to 15, please select correct choice (Yes / No)**

| No | Yes | Items |
| --- | --- | --- |
|  |  | **1. Can brucellosis be transmitted from an animal to a human?**  **2. Can brucellosis be transmitted from a sheep or a goat to a human?**  **3. Can brucellosis be transmitted from a cow to a human?**  **4. Is brucellosis transmitted from a person to another person?**  **5. Can brucellosis be transmitted from skin contact with an infected animal?**  **6. Can brucellosis be transmitted from touching the aborted fetus and placenta of an infected animal?**  **7. Can wearing gloves prevent getting brucellosis when you contact uterine secretions of dead animals?**  **8. Does the brucellosis spread in the environment by animal’s urine?**  **-9. Does the brucellosis spread in the environment by animals’ fetus and placenta?**  **10. Does the brucellosis spread in the environment by wool?**  **11. Do you disinfect the place of abortion when an abortion happens?**  **12. Do you think a dog can eat an aborted fetus?**  **13. Do you think the aborted fetus needs to be buried?**  **14. Is brucellosis a preventable disease?**  **15. Should the area be disinfected after an abortion was occurred?** |

**Responding to questions from 16 to 24, please select correct choice (Yes / No)**

| No | Yes | Items |
| --- | --- | --- |
|  |  | **16. Can brucellosis be transmitted through consumption of infected milk and dairy products?**  **17. Can brucellosis be transmitted from semi-cooked meat?**  **18. Can brucellosis be transmitted from Breathing into infected stalls of animals?**  **19. Can washing milking dishes prevent getting brucellosis?**  **20. Can boiling milk prevent getting brucellosis?**  **21. Can keeping cheese in salty water for 2 months before consumption prevent getting brucellosis?**  **22. Does the brucellosis spread in the environment by milks of infected livestock?**  **23. Does the brucellosis spread in the environment by meat of infected livestock?**  **24. Do you ask a veterinarian to help for an animal’s abortion?** |

**Responding to questions from 25 to 34, please select the correct choice**

| No | Yes | Items |
| --- | --- | --- |
|  |  | **25. Can vaccination of livestock prevent the Malta fever among humans?**  **26**. **Can brucellosis be prevented by vaccination of livestock?**  **27. Is the cost of vaccinating animals high for you?**  **28. Do you have access to livestock vaccination services?**  **29. Does the veterinary organization offer timely vaccination services for your animals?**  **30. Do the veterinarians encourage you to vaccinate your livestock?**  **31. Do the health personnel encourage you to vaccinate your livestock?**  **32. In which season should brucellosis vaccine be injected?**  Spring &Summer   Autumn & Winter  **33. How often should the brucellosis vaccine be repeated?**  Every year  No need to repeat     **34. At what age should livestock be vaccinated against brucellosis?**  At the age of three to six months One-year-old  |

**Complete the following sentences by the proper statements.**

|  |  | Items |
| --- | --- | --- |
|  |  | **35. After vaccination against brucellosis,**  Growth of livestock increases and they become healthier  Growth of livestock reduces and they become weaker  **36. After brucellosis vaccination,**  Brucellosis will not occur  Animals can get brucellosis again  |

**Responding to questions 37 to 45, please choose one of the options (Strongly Agree, Agree, Moderately agree, Disagree or strongly disagree)**

| **Item** | **Strongly Agree** | **Agree** | **Moderately agree** | **Disagree** | **Strongly disagree** |
| --- | --- | --- | --- | --- | --- |
| **37. There will be no abortion, if the animals are vaccinated.** |  |  |  |  |  |
| **38. Livestock breeders should ask a veterinarian to examine their livestock.** |  |  |  |  |  |
| **39. I will not get brucellosis, if I touch**  **Vaccinated animals’ milk, urine, placenta and fetus.** |  |  |  |  |  |
| **40. As vaccination is time-consuming, I prefer getting brucellosis instead of vaccinating my animals.** |  |  |  |  |  |
| **41. Animal vaccination is a very difficult process.** |  |  |  |  |  |
| **42. If I get brucellosis, I will be unable to work for a long time.** |  |  |  |  |  |
| **43. If I do not vaccinate the animals, my family and I will get brucellosis.** |  |  |  |  |  |
| **44. If I vaccinate my livestock, people will not get brucellosis with consumption of my dairy products.** |  |  |  |  |  |
| **45. I may still be in danger of getting brucellosis, even if I do preventative measures.** |  |  |  |  |  |

**Responding to questions 46 to 53, choose one of the options (Always, Often,**

**Sometimes, Rarely or Never).**

| **Question** | **Always** | **Often** | **Sometimes** | **Rarely** | **Never** |
| --- | --- | --- | --- | --- | --- |
| **46. I keep records and documents of medical history and vaccination of my livestock in a proper place.** |  |  |  |  |  |
| **47. I have experienced abortion among my livestock.** |  |  |  |  |  |
| **48. I always keep an eye on the cold chain about vaccines being injected into my cattle.** |  |  |  |  |  |
| **49. I vaccinate my livestock at the proper and recommended time in a year or season.** |  |  |  |  |  |
| **50. Every year, I vaccinate my livestock against brucellosis and I have done it for many years.** |  |  |  |  |  |
| **51. My livestock are in contact with other non-vaccinated livestock.** |  |  |  |  |  |
| **52. When I buy a new livestock, I am curious to know about their vaccination history.** |  |  |  |  |  |
| **53. When I buy a new livestock, I ask a vet to examine my animal.** |  |  |  |  |  |
